# Characterization of Microstructures and Tensile Properties of Recycled Al-Si-Cu-Fe-Mn Alloys with Individual and Combined Addition of Titanium and Cerium

**DOI:** 10.1155/2018/3472743

**Published:** 2018-12-17

**Authors:** Kang Wang, Peng Tang, Yi Huang, Yanjun Zhao, Wenfang Li, Jun Tian

**Affiliations:** ^1^School of Mechanical Engineering, Dongguan University of Technology, Dongguan, 523000 Guangdong, China; ^2^Guangxi Key Laboratory of Processing for Non-Ferrous Metals and Featured Materials, Nanning, 530004 Guangxi, China; ^3^School of Resources, Environment and Materials, Guangxi University, Nanning, 530004 Guangxi, China

## Abstract

Individual and combined addition of Ti and Ce on the recycled Al-Si-Cu-Fe-Mn alloy was conducted. The microstructures and tensile properties of these fabricated alloys were investigated. In the case of Ti or Ce which was individually added, the added amount was ranging from 0.03 wt.% to 0.09 wt.%. The combined addition of Ti and Ce was set at the ratios of 1 : 1, 1 : 3, and 3 : 1 with a total amount of 0.12 wt.%. Microstructures and phases of these alloys were investigated by using an optical microscope, X-ray diffraction testing, and SEM coupled with EDS. The morphologies of these alloys were quantified by analyzing the SDAS value, length of secondary phases, and phases' distribution uniformity. Tensile testing was carried out for understanding the strengthen effect of the modification process. Results show that the addition of Ce was favorable to the strength and % elongation because the coarse needle-like phase and the polyhedral phase were effectively refined. Their SDAS values and distribution factor were remarkably declined with the increase of the Ce level. The Ti addition could also refine the secondary phases and SDAS values. But its effect was not as prominent as the addition of Ce. Combined addition of Ti and Ce elements at the ratio of 1 : 3 resulted in the samples reaching maximum comprehensive tensile properties. In this case, the short needle-like phase was uniformly distributed in the microstructure. Few polyhedral phases could be found in the Al-Si-Cu-Fe-Mn matrix. The strengthening of these fabricated materials was due to the grain refinement for *α*-Al and modification for coarse secondary phases. In addition, distribution uniformity of secondary phases was also changed by their modification effects.

## 1. Introduction

In recent years, many kinds of cast Al alloys have been used in the engineering field due to their high fluidity, low casting shrinkage rate, good corrosion resistance, and relatively high strength [1–3]. Since the eutectic Al-Si and Al-Cu phases are favorable to the alloy's strength and the subsequent heat treatment process, they have been widely applied in the structural parts of the aerospace and auto industries [4, 5].

Instead of producing the primary Al-Si alloys from bauxite ores, obtaining these alloys from recycling the aluminum scrap (such as the beverage cans and abandoned automotive parts) is accepted by the industry. It has been encouraged for the purpose of energy saving and cost reduction [6]. The Al scrap is abundantly available. But the content of iron in aluminum scrap is relatively high. According to relevant references [7, 8], the iron in Al-Si alloys will lead to the formation of a coarse needle-like *β*-AlFeSi phase when its concentration exceeds about 1%. The removal of iron from the Al matrix is widely taken as one of the most valid methods for the enhancement of mechanical properties [9, 10]. However, the iron is also necessary to some extent in the Al alloys. For instance, the trace iron in Al alloys can facilitate the separation of casting parts from the die casting molds [8].

In order to promote the mechanical and tribological properties of recycled Al alloys, more and more researches are focused on changing the morphologies of needle-like iron phases by adding the neutralized elements (i.e., Ti, Sr, B, Ce, Cr, etc.) [7, 11–13]. Among these neutralized elements, the manganese has been widely added into the secondary Al alloys due to the refinement and modification effect on needle-like phases [14, 15]. Although the morphologies of needle-like phases could be remarkably changed by using manganese, the promotion of their mechanical properties were not prominent enough [11, 13]. Several recent studies [16, 17] suggested that the mechanical properties of cast aluminum alloys could be further enhanced via simultaneously adding two kinds of neutralized elements to refine coarse phases. Farahany et al. [18] studied the interaction of Sr and Bi elements on the mechanical properties of Al-Si-Cu-Fe-Zn alloy. They pointed out that the combined addition of trace Bi and Sr elements in a certain proportion could remarkably change the shape of needle-like Al-Si-Fe phases in the matrix.

In our previous study [19], we found that the addition of titanium could modify the *β*-Al_5_FeSi. Several studies [20, 21] also suggested that titanium was favorable to the refinement of needle-like secondary phases. In addition, it is noteworthy that the cerium could effectively modify the needle-like ferrous phase in the alloy [11, 22–24]. At present, the manganese has been widely added in many commercial aluminum alloys with relatively high Fe content (>1 wt.%) for effectively reducing the dimension of needle-like iron phases [25, 26]. To further strengthen the materials, individually adding the trace elements into the molten alloy can no longer satisfy the industrial demand [27, 28].

In this work, the manganese was added into the recycled Al-Si-Cu-Fe alloy in advance to modify the needle-like iron phases. Effects of individual and combined addition of titanium and cerium on the microstructures and strengths of these Al alloys were investigated. The methods of dimensional measurement [29] for the microstructures were carried out. Besides, it is noticed that the distribution uniformity of these secondary phases would also be changed after these phases were modified. But only a few researchers have analyzed the relationship between the microstructures and mechanical properties by taking the distribution uniformity factor (*F*_d_) into account [30, 31]. In order to know the effect of added elements on the feature of microstructures, in this paper, the dimensions and distribution uniformity of secondary phases were both quantified.

## 2. Experimental Procedures

### 2.1. Alloy Preparation

The Al-Si-Fe-Cu-Mn-based alloy with relatively high Fe content (with 2.12 wt.% iron content, manufactured by Kinbon Nonferrous Alloy Metal Co., Guangzhou, China) was prepared by using secondary Al alloy. The resources of titanium and cerium were from the Al-5Ti-C and Al-10%Ce master alloys. These master alloys were supplied from Shandong Al&Mg Melt Technology Co. Ltd. The chemical compositions of the experimental alloys are listed in Table 1 (measured by the direct-reading spectrometer, SPECTROLAB/M11, Germany).

The Al-Si-Cu-Fe-Mn alloy was deposited in an Al_2_O_3_ crucible and melted in an electric resistance furnace. After keeping the Al melt in the crucible at 740°C for 30 min, the C_2_Cl_6_ degassing tablet was added. Then, the molten alloy was stabilized by holding at 720°C for 10 min. Different levels of titanium (Ti) and cerium (Ce) in the form of master alloys were wrapped with aluminum foil and immersed into the melt. After throwing the wrapped master alloys into the melt, a cap linked with a stick, which were both made of graphite, was immersed into the melt to guarantee that these master alloys were completely immersed. The dosage of Ti and Ce elements in the based Al-Si-Cu-Fe-Mn alloy is given in Table 2. The individual additional amounts of these two elements were both ranged from 0.03 wt.% to 0.09 wt.%. In the case of combined addition of titanium and cerium, the total amount of them was 0.12 wt.%. As listed in Table 2, the Ti : Ce ratios were set as 1 : 1, 1 : 3, and 3 : 1, respectively. The melts were kept in the furnace for about 30 min for dissolution and homogenization. Thereafter, the oxide layer on the surface of the molten alloy was skimmed and carefully poured into a permanent mold preheated to 200°C. Samples for metallurgical analysis and tensile testing were machined out from these cast ingots and cooled down at room temperature.

### 2.2. Observation and Quantification for the Microstructure

In order to confirm the effect of the added trace elements on the microstructures of the alloys, the observation and quantification for the optical microstructure were carried out. These samples were machined out from the same position of the cast ingot. After grinding and polishing operation, they were etched by using the solution composed of 0.5 ml hydrofluoric acid and 100 ml H_2_O. Metallographic analysis was conducted by the optical microscope (Zeiss/Observer. A1, Germany) and the scanning electron microscope (Phenom/Nano-430, USA) coupled with EDS device. The X-ray diffraction analysis (XRD, PANalytical/X'Pert Pro MRD, Netherlands) was conducted for determining the phases in the fabricated samples.

Refinement and the modifying effects of the Fe-rich phases were assessed by microstructure quantification. For this purpose, the microstructure images of each fabricated alloy were obtained from five different randomly selected areas. At least 50 statistical samples were also randomly determined in each view field. Similarly with the relevant literatures [29, 32], the secondary dendrite arm spacing (SDAS) values and lengths of secondary phases were measured to assess the modification and refinement effects in these five view fields:

(1) $$\begin{array}{c} Mean\;length=\frac{1}{m}\overset{m}{\underset{j=1}{\sum }}{\left(\frac{1}{n}\overset{n}{\underset{i=1}{\sum }}L_{i}\right)}_{j}, \end{array}$$

where *L*_*i*_ is the area and aspect ratio of an arbitrary modified secondary phase in the microstructure, *n* is the number of particles measured in a view field, and *m* is the number of the fields for quantification. In this case, the approximate SDAS values were statistically analyzed via nanomeasurement software. The *n* value is determined as 50, and *m* is 5.

**Figure 1:**
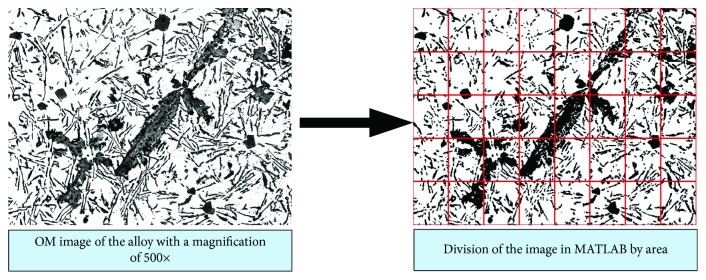
The process of distribution factor (*F*_d_) determination.

The added elements in these aluminum alloys might not only cause the refinement and modification effect of the phases but also lead to different distribution uniformities of phases in the matrix. Hence, the tessellation method [31] was carried out for the quantification of distribution uniformity. As shown in Figure 1, the microstructure image (with a dimension of 2088 × 1550 pixel^2^) was imported into MATLAB program.

As seen from Figure 1, the obtained microstructure image was evenly divided into 40 units by area. Here, the dimension of the whole area is represented by *A*_*f*_, the total area of secondary phases in each separated unit is symbolized by *A*_*i*_ (the corner mark “*i*” is used for labeling each arbitrary secondary phase in a specific unit), the amount of the secondary phase is counted by the program and given as *n*, and the amount of the divided unit is symbolized by *S* (i.e., *S* = 40 in this situation). The distribution uniformity of the secondary phases in a picture can be determined by the distribution factor (*F*_d_) given as follows:

(2) $$\begin{array}{c} F_{d}=\frac{\sqrt{\left(1/\left(S-1\right)\right)\sum_{i=1}^{n}{\left(A_{i}-\sum_{i=1}^{n}A_{i}\right)}^{2}}}{A_{f}}. \end{array}$$

According to the theory [31], the lower was the *F*_d_ value, the more uniform the secondary phases were distributed in the alloy's matrix. In this study, five view fields taken from different regions in one image were carried out for this analysis. The mean value of *F*_d_ was taken as the final result to reflect the degree of distribution uniformity of secondary phases.

### 2.3. Mechanical Characterization and Quality Index

**Figure 2:**
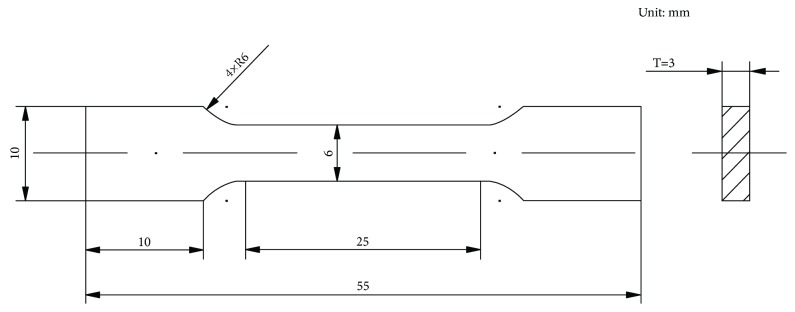
Dimensions of the tensile testing sample.

All of the samples for tensile testing were taken from the center of the ingots. The tensile testing was conducted by using the computerized testing machine (Shimadzu/AG­X, Japan) at room temperature (25°C). The yield strength (YS), ultimate tensile strength (UTS), and ductility were obtained at a strain rate of 1 mm/min. According to ASTM E8M-04 standard (subsize sample, as presented in Figure 2), five tensile testing samples were machined out for each alloy. The fracture surfaces of these specimens were further investigated via the SEM.

## 3. Results

### 3.1. Analysis of the *α*-Al Grains

**Figure 3:**
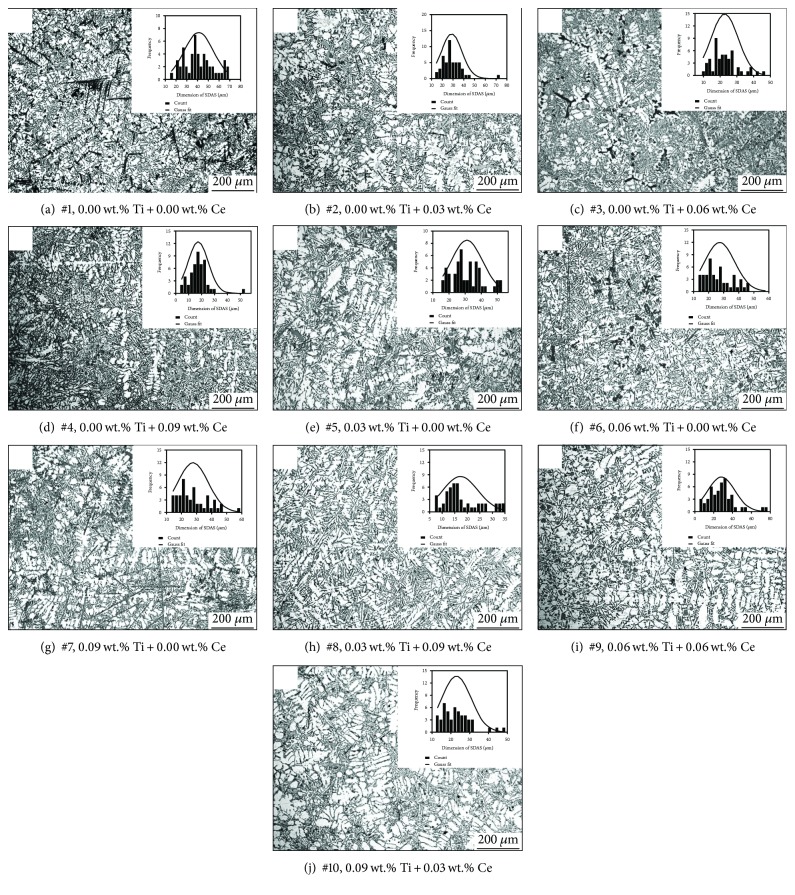
Microstructure of the samples with different added amounts of Ti and Ce.

**Figure 4:**
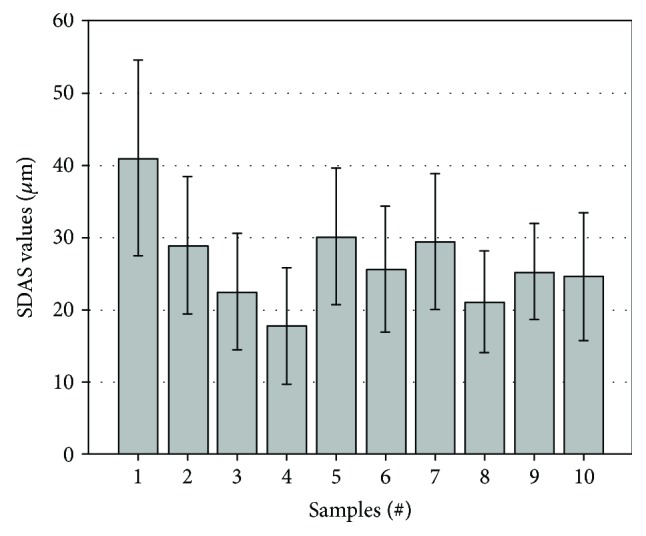
The mean SDAS value of the alloys with different amounts of Ti and Ce.

In order to confirm the refinement effect of added trace elements on *α*-Al grains, secondary dendrite arm spacing (SDAS) values of fabricated samples are statistically measured and given in Figure 3. The mean SDAS values of these alloys are concluded in Figure 4. From Figure 3(a), without any addition of trace elements, the secondary phases and the *α*-Al grains in the Al-Si-Cu-Fe-Mn-based alloy are extremely coarse. The cerium addition in the based alloy leads to a significant change of microstructure even though its dosage is only 0.03 wt.%. As seen in Figure 3(b), by increasing the cerium amount, the coarse needle-like phases are shortened and then *α*-Al grains are refined. With the increase of added amount of cerium from 0.03 wt.% to 0.09 wt.% (Figures 3(c) and 3(d)), the size of *α*-Al grains is decreased continuously. It is also noted that the dimensions of secondary phases decrease with the increase of added amounts of cerium. Figures 3(e)–3(g) are the microstructure images of the based alloys with titanium added. When the amount of titanium increases from 0.03 wt.% to 0.06 wt.%, the SDAS value is decreased. But it increases again when the titanium amount increases to 0.09 wt.%. From Figure 4, in the situation in which trace elements are individually added, it is found that the refinement effect of cerium for *α*-Al is more prominent than the titanium. The microstructures of the alloys with the combined addition of Ti and Ce elements are given in Figures 3(h)–3(j). Obviously, the SDAS value and the length of the secondary phases reach the maximum value when the added amount of titanium and cerium is determined at 0.03 wt.% and 0.09 wt.%, respectively (adding proportion of Ti : Ce at 1 : 3). However, the SDAS value increases while increasing the titanium level.

### 3.2. Insight of the Secondary Phases

**Figure 5:**
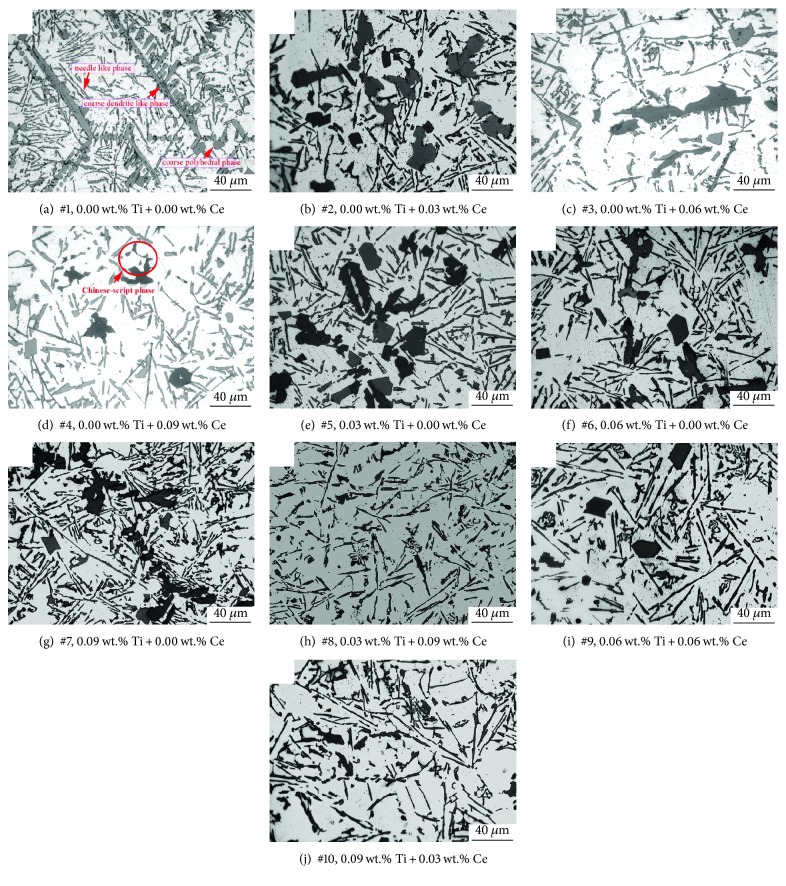
The microstructure of the samples with different added amounts of Ti and Ce.

Figure 5 displays the microstructures of the Al-Si-Cu-Fe-Mn alloys with different amounts of titanium and cerium at a higher magnification.

As shown, the formed secondary phases with different shapes after adding different levels of Ti and Ce can be observed.  Figure 5(a) displays the microstructure of Al-Si-Cu-Fe-Mn-based alloy. It can be seen that the secondary phases with needle-like shape, coarse polyhedral shape, and coarse dendrite-like shape are existed in the microstructure. Figures 5(b)–5(d) show the microstructures with different amounts of cerium individually added. It is clear that the coarse dendrite-like phase cannot be found in this sample when the added amount of cerium is 0.03 wt.%. Compared with Figure 5(a), the polyhedral phase size is larger in this case. With the dosage of cerium increases to 0.06 wt.%, the amount of coarse polyhedral phases decreases (Figure 5(c)). Meanwhile, the phase with Chinese-script shape is revealed. From Figure 5(d), it is found that the polyhedral phase is fine when the dosage of cerium is increased to 0.09 wt.%. In this case, the amount of Chinese-script phase still exists in the view field.

The microstructures of the Al-Si-Cu-Fe-Mn alloys with different individual added amounts of titanium are displayed in Figures 5(e)–5(g). Compared with the microstructure of the based alloy, the coarse dendrite-like phase is absent in the alloy when the dosage of titanium is set at 0.03 wt.% (see details in Figure 5(e)). In this case, the polyhedral phase becomes coarser, but the change of the needle-like phase is not prominent. When the individual addition of titanium increases to 0.06 wt.%, all of the secondary phases (as shown in Figure 5(f)) are finer. With the titanium dosage increases to 0.09 wt.%, it is found that the dimension of the needle-like phase becomes coarser again (Figure 5(g)).

Figures 5(h)–5(j) show the microstructure of the based alloy with the combined addition of titanium and cerium. Compared with the alloys with individual additions of titanium or cerium, fewer coarse polyhedral phases can be found when the combined addition of Ti and Ce is at 0.03 wt.% and 0.09 wt.%, respectively. Among these microstructures, the refinement effect of the needle-like phase is prominent in sample #8 (Figure 5(h)). When the added amounts of titanium and cerium are both 0.06 wt.%, some coarse polyhedral phases reveal again (see details in Figure 5(i)). From Figure 5(j), it is found that the size of the polyhedral phase is prominently decreased, but the needle-like phase is coarser. As far as the microstructure of samples #8~#10, several Chinese-script phases with relatively small size can also be found.

**Figure 6:**
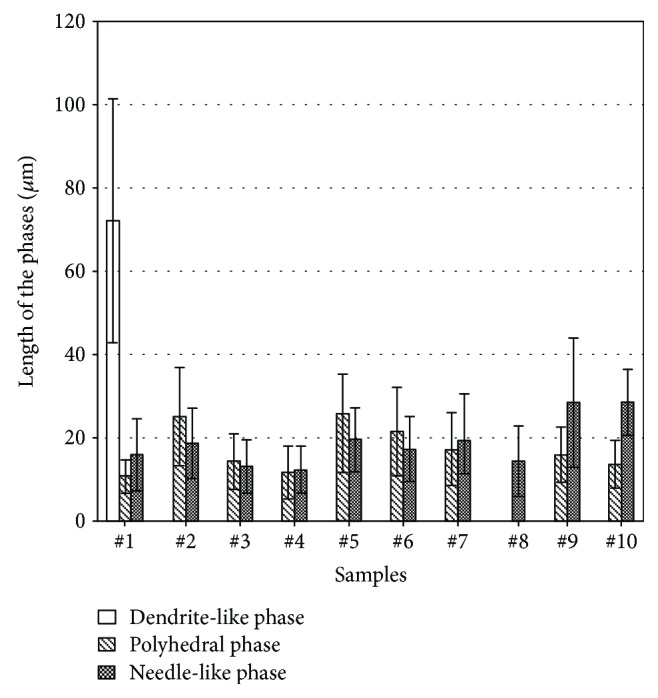
The length of the secondary phase in the samples.

The length of secondary phases is statistically measured and provided in Figure 6. It can be seen that the length of dendrite-like phases of alloys with cerium added is only about 1/4 to 1/3 of the based alloy. The minimum length of needle-like phase is revealed in sample #4. This sample has the most prominent comprehensive refinement effect of secondary phases. In the situation of titanium and cerium simultaneously added, the length of needle-like phases in sample #8 is the lowest (with the composition of Ti and Ce elements which was set at the proportion of 1 : 3). However, it still possesses a larger needle-like phase compared with samples with cerium individually added (samples #3 and #4), but slightly lower than that in the alloys with titanium individually added. From sample #8, the polyhedral phase is vanished. Polyhedral phase reappears when the dose of titanium increases (see details of the data about samples #9 and #10). The sizes of these secondary phases also are increasing.

### 3.3. Phase Identification of the Intermetallic Phases

**Figure 7:**
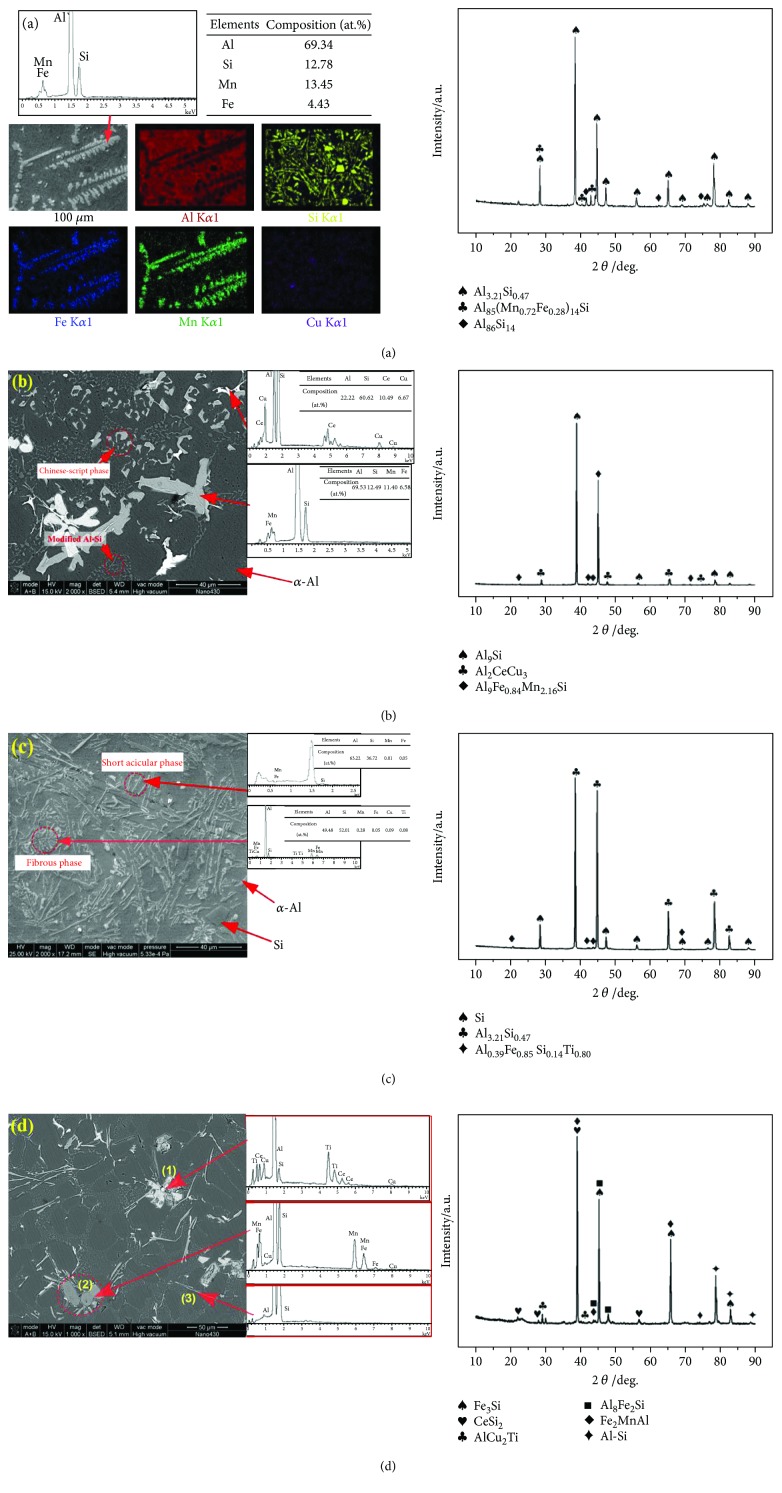
Phase identification of the fabricated samples by using SEM/EDS and XRD: (a) the Al-Si-Cu-Fe-Mn alloy, (b) the alloy with Ce, (c) the alloy with Ti, (d) the alloy with Ce and Ti.

The phases in the fabricated alloys have been identified via EDS and X-ray diffraction testing.  Figure 7(a) shows the SEM image and EDS testing (mapping mode) of the based alloy. It suggests that dendrite-like, polyhedral, and needle-like phases are Al-Fe-Mn phase, silicon phase, and Al-Si eutectic phase [6, 15], respectively. It is worth noting that the silicon amount in the mother alloy is only 10.9%, which is less than the hypereutectic composition. The existent of polyhedral silicon in this alloy is due to the segregation of silicon during the solidification process of aluminum. This phenomenon has also been reported by several references [33, 34].

The EDS testing results given in Figure 7(a) indicate that the coarse dendrite-like phase is mainly composed of Fe and Mn elements. The XRD analysis suggests that the dendrite-like phase might be the Al_85_(Mn_0.72_Fe_0.28_)_14_Si phase. In addition, the Al_3.21_Si_0.47_ (eutectic Al-Si) and the Al_86_Fe_14_ existed. In the situation of Al-Si-Cu-Fe-Mn alloy with cerium added (Figure 7(b)), the phases with different grayscale images can be observed. After EDS and XRD analysis, the bright phase might be the Al_2_CeCu_3_. The phase with Chinese-script shape is Al_9_Fe_0.84_Mn_2.16_Si. The fine fibrous phase distributed in the matrix is the modified eutectic Al-Si (identified as Al_9_Si).  Figure 7(c) shows the SEM micrograph of the Al-Si-Mn-Fe alloy with titanium added. The SEM microstructure displays the fibrous phase and short acicular phase. According to the XRD pattern, they are the Al_0.39_Fe_0.85_Si_0.14_Ti_0.80_ and Al_3.21_Si_0.47_ phases, respectively.

From the XRD results given in Figure 7(d), it is found that five kinds of compounds are formed in the alloy with titanium and cerium simultaneously added. Three kinds of typical phases are marked as “(1),” “(2),” and “(3)” in the SEM microstructure image, and the compositions of these secondary phases are provided in Table 3. Since titanium and cerium can be detected on secondary phase “(1),” it might be the mixture of AlCu_2_Ti and CeSi_2_. The elements of Al, Si, Mn, Fe, and Cu can be detected in the region, which labeled as “(2).” It is noticed that the concentration of copper in this secondary phase is extremely low. Hence, it might be the Fe_3_Si, Al_8_Fe_2_Si, and Fe_2_MnAl phases which precipitate from the Al matrix with Cu element dissolved. The needle-like phase in the SEM image is marked as “(3).” By analyzing the EDS and XRD results, this phase can be identified as Al-Si eutectic.

### 3.4. Mechanical Properties and Fracture Morphology

**Figure 8:**
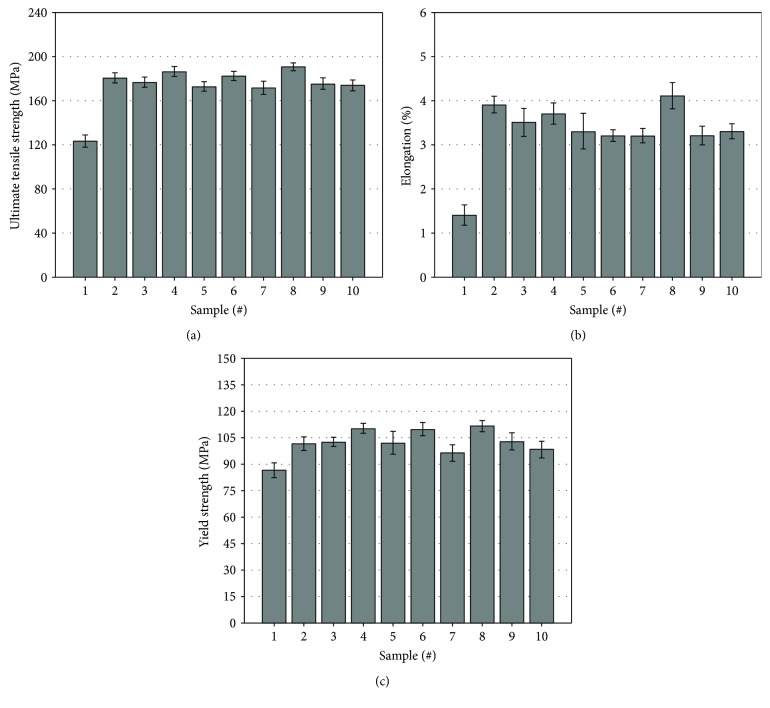
Tensile properties of these alloys. (a) Ultimate tensile strength. (b) Percentage of elongation. (c) Yield strength.

The tensile properties containing yield strength (YS), ultimate tensile strength (UTS), and % elongation of the fabricated alloys are presented in Figure 8.

From Figure 8, it is clear that additions of titanium and cerium can effectively promote the comprehensive tensile properties of the based alloys. After adding these elements, the ultimate tensile strength (UTS), percentage elongation, and the yield strength (YS) are all increased.

When the cerium is individually added into the based alloy with amounts from 0.03 to 0.09 wt.% (samples #2~#4), it is found that the YS value of the alloys is linearly increased. But the UTS and % elongation values decrease at first, then they increase again. The UTS value of the based alloy with cerium added reaches the maximum at sample #4 (186.3 MPa), which is 51.2% higher than that of the based alloy. But the % elongation of sample #4 is not higher than sample #2 which is nearly three times higher than that of the based alloy. It is worth noting that the UTS and YS values of sample #4 are the second highest among all of the samples.

When the titanium is individually added into the based alloy with amounts from 0.03 to 0.09 wt.%, it is found that the % elongation value of these alloys is linearly decreased. The variation trends of the UTS and YS values in these three samples are the same. It is found that the sample #6 possesses the third highest UTS and YS values. The UTS of sample #6 is about 48% higher than that of the based alloy, and it is only 4 MPa lower than that of sample #4. The difference of YS values between sample #6 and sample #4 is only 0.2 MPa.

It is worth noting that the strengthen effect from the combined addition of cerium and titanium is more prominent. When titanium and cerium are added at a ratio of 1 : 3 (sample #8, Ti: 0.03 wt.%, Ce: 0.09 wt.%), the UTS value reaches the maximum among all of these experimental samples. It is 54.6% higher than the based alloy. Compared with the other samples, the % elongation and YS values also reach the maximum. But when the addition of Ti element exceeds 0.03 wt.%, the strength and elongation values are all decreased. The UTS and % elongation values are declining with the dosage augmentation of titanium. In these situations of simultaneously adding titanium and cerium, the best strengthening effect of the alloy revealed at the combined addition of titanium and cerium at a ratio of 1 : 3.

**Figure 9:**
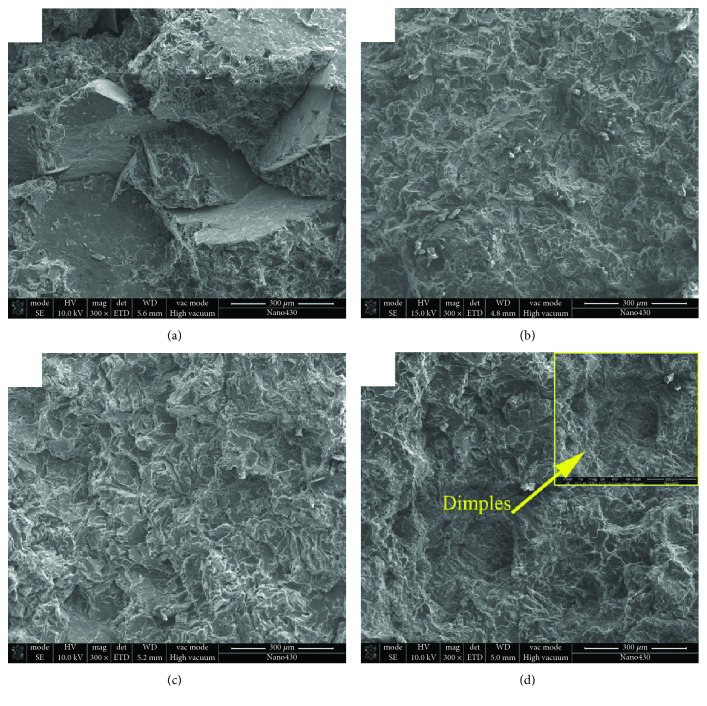
Fracture patterns of Al-Si-Cu-Fe-Mn alloys with different added amounts of Ti and Ce: (a) the based alloy, (b) Ce: 0.09 wt.%, (c) Ti: 0.06 wt.%, (d) Ti: 0.03 wt.% + Ce: 0.09 wt.%.

Several typical fracture surfaces of the fabricated alloys are selected and given in Figure 9.  Figure 9(a) shows the fracture surface of the based alloy. The coarse cleavage surfaces can be found in this pattern, which indicates that the destruction of this alloy is caused by the intergranular crack. From the results of microstructure analysis, it is found that the mean SDAS value of the based alloy (Figure 4) reaches the maximum, which has a good agreement with the coarse cleavage surface in this pattern. Compared with Figure 9(a), it is found that the cleavage surfaces in Figure 9(b) are smaller. It is due to the effect of cerium addition on the decrease of SDAS values. Besides, several fine particles can also be observed in this fracture pattern. As far as the samples of the based alloys with titanium additions (Figure 9(c)), it is clear that the cleavage surface is larger than that with cerium addition. It is corresponding to the SDAS results given in Figure 4.  Figure 9(d) shows the fracture surface with the finer cleavage surface. It resulted from the cracks which penetrate through the finer grains in this material, which supports the higher YS and % elongation values of sample #8 (see details in Figure 8). The fracture pattern with higher magnification of sample #8 is provided at the top right-hand corner of Figure 9(d). Several small dimples can be observed in this micrograph.

### 3.5. Distribution Uniformity of Secondary Phases

**Figure 10:**
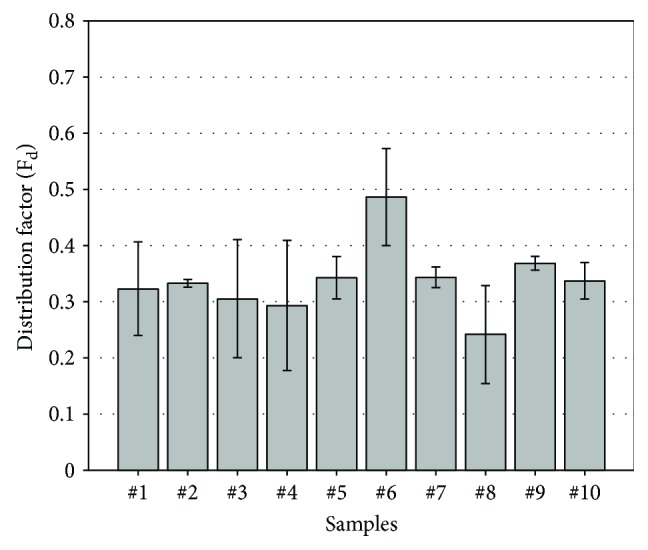
Distribution factors of secondary phases in these alloys.

Observed from the microstructures given in Figures 3 and 5, the SDAS values and dimension of secondary phases are changed with the additions of elements. Meanwhile, it is worth noting that the distribution uniformity of secondary phases is also varied. According to the microstructure images (magnification of 500x) and the method introduced in Figure 1, the distribution factors (*F*_d_, calculated by (2)) are concluded in Figure 10.

The result indicates that the highest *F*_d_ reveals at sample #6, implying that the distribution of secondary phases in the matrix is relatively nonuniform. In the case when titanium or cerium is added, this phenomenon supports the low UTS, % elongation, and YS values of sample #6. The samples #8 and #4 have the lowest and the second lowest *F*_d_ value, respectively. They correlate with the relatively high strength displayed in Figure 8. It also suggests that the addition of 0.03 wt.% titanium in the sample with 0.09 wt.% cerium is helpful to improve the distribution uniformity of secondary phases in aluminum matrix. Because the refined coarse phases in the microstructure lead to the decrease of the *F*_d_ value by balancing the areas in every quantification unit.

## 4. Discussion

### 4.1. Effect of Ti and Ce

From the EDS investigation in this study (see details in Figure 7), it is known that the coarse dendrite-like phase and the needle-like phase are mainly composed of aluminum, silicon, iron, and manganese. The compounds formed in the Al-Fe-Mn-Si system under equilibrium condition have been summarized by Raghavan [35]. In this study, these samples have been all cooled down at room temperature. The stoichiometries of modified and refined phases have been determined via EDS testing.

While analyzing the microstructures and the quantitative results of phases' dimension, it is found that the addition of cerium on refinement of the secondary phases and SDAS value is prominent. Instead of revealing the coarse phases, the finer fibrous phases are observed after the cerium or titanium was added. This phenomenon has been supported by Fan et al. [36] that the size of Chinese-script phase and the *α*-Al grain size will decrease with the increase of cerium addition. The modifying effect of cerium on decreasing the size and shape of the primary Si phase has also been proven [37]. Meanwhile, many researchers have mentioned that the addition of titanium would lead to the formation of the Al_3_Ti phase, which was the heterogeneous nucleus for *α*-Al grain. The addition of Al-Ti-C might lead to the formation of Al_4_C_3_ in these aluminum alloys, which has also been taken as the nucleation core for the phases with silicon contained [38].

The modification and refinement behaviors of cerium and titanium are due to (1) impurity-induced twining [39], (2) suppression of nucleation temperature of secondary phases [40], and (3) heterogeneous nucleation [41]. In this study, the silicon content in the mother alloy is about 11%. The relatively coarse phases in the matrix are formed by high silicon content to some extent. The difference of atomic radii between added elements and the silicon might be the dominant factor for the modification. In consideration of the impurity-induced twining theory, the modifying effect of Ti and Ce in the Al-Si-Cu-Fe-Mn alloys depends on the atomic radius ratio of the elements. The ideal atomic radius ratio, reported by Lu and Hellawell, [39] was 1.646. According to the data of atomic radii [42], the atomic radius ratio of Ti (*r*_Ti_/*r*_Si_) is 1.239 and the atomic radium ratio of Ce (*r*_Ce_/*r*_Si_) is 1.56. It is obvious that the radium ratio of Ce is very close to the ideal condition of the modification. Hence, the addition of cerium in Al-Si-Cu-Fe-Mn alloy has a better modification effect than the addition of titanium.

As far as the samples with cerium individually added (samples #2~#4), it is found that the SDAS values, sizes of secondary phases, and the *F*_d_ values decrease with the increase of cerium dosages. The modified secondary phases possess smaller size. With the increase of cerium amount in the alloy, the variation between polyhedral phase and needle-like phase is decreased. It leads to the decrease trend of *F*_d_ value among samples #2~#4. In addition, the finer secondary phases have a larger specific area in the aluminum alloy. During the alloys' solidification process, the finer secondary phases would hinder the growth of *α*-Al grain or provide more heterogeneous nucleation cores for *α*-Al grain, which achieved the decrease of SDAS values.

As far as the samples with titanium individually added (samples #5~#7), it is found that the modifying effect is less prominent than the mother alloy with cerium individually added. The dimensions of secondary phases are decreased with the increase of titanium dosages. But the minimum SDAS value reveals at sample #6 (with 0.06 wt.% Ti added). We have also reported that the higher added amount of titanium will lead to the recession of the refinement effect of *α*-Al [19]. From Figure 6, it can be seen that the dimension difference between polyhedral phase and needle-like phase is still prominent in samples #5~#7. It might be the reason for the high *F*_d_ value of the samples with different individually added titanium.

In the situation of samples with the combined addition of Ti and Ce, the changes of their microstructures are obvious. The polyhedral silicon phase can rarely be found in sample #8 in which the ratio of Ti : Ce is 1 : 3. The fine needle-like phase is uniformly distributed in the matrix, leading to the relatively lower *F*_d_ value of this sample. With the increase of titanium dosage, a few polyhedral phases with small size are shown up again and the length of needle-like phase becomes higher.

From the above analysis, it can be concluded that both the polyhedral phase and the needle-like phase can be refined by the addition of cerium. The titanium can also modify and refine these two kinds of phases, but its effect is not as remarkable as cerium. The modification and refinement effects are also prominent when titanium and cerium are simultaneously added at the ratio of 1 : 3. The reason of this phenomenon might be Ti and Ce could react with the elements such as Si, Cu, and Fe, forming multiple secondary phases and avoiding the generation of coarse phases. When the addition of the ratio of Ti : Ce increases, more titanium could be provided to form these secondary phases. In this case, the refinement effect of cerium is weakening. Hence, the dimensions of secondary phases are increased.

### 4.2. Strengthen Mechanism of Al-Si-Cu-Fe-Mn Alloy

The grain size, dimension, and distribution uniformity of secondary phases are varied with different trace elements added. After comparison, it is found that Al-Si-Cu-Fe-Mn alloys with cerium added possess relatively lower SDAS value and finer secondary phases. The modification for coarse needle-like and polyhedral phases is favorable to avoid the tendency of stress concentration in the microstructures. The finer *α*-Al grains possess a larger specific surface area of the grain boundaries, which is beneficial to dislocation slipping [43]. Meanwhile, the *F*_d_ values of samples #3 and #4 are relatively low, which contributes in preventing the stress concentration and defect formation in the microstructure [44, 45].

In the situation of alloys with titanium added, the decrease of SDAS value is not as remarkable as the alloy with cerium added. The distribution of secondary phases in the alloys with titanium is less homogeneous than that in the alloys with cerium added. Among these three samples, the sample #6 possesses the lowest SDAS value but its *F*_d_ value is especially high. From Figure 8, its % elongation is low, but its strength is higher than the other samples while titanium is individually added. It suggests that the distribution uniformity of secondary phases in the Al-Si-Cu-Fe-Mn alloy will prominently impact its ductility.

Samples #8~#10 are the alloys with cerium and titanium simultaneously added. It is found that the tensile properties of samples #9 and #10 are poor. But the sample #8 possesses the most attractive tensile properties. Since the % elongation of sample #8 is relatively high, several dimples have existed on the fracture surface (Figure 9(d)). In addition, the ultimate tensile and yield strength of sample #8 are also higher than the other samples. After simultaneously modified by titanium and cerium, this sample possesses a low SDAS value and fine and uniformly distributed secondary phases, which are the main factors for the good comprehensive tensile properties.

## 5. Conclusions

The effects of adding cerium and titanium on microstructures and mechanical properties of the Al-Si-Cu-Fe-Mn alloys were investigated. The following conclusions can be drawn:

1. Individual and combined addition of titanium and cerium led to the refinement of secondary phases and *α*-Al grains. Besides, the distribution uniformity of secondary phases in the matrix was also improved
2. The added amount of cerium at 0.09 wt.% could effectively refine the *α*-Al grain and modify the needle-like phases
3. The added amount of titanium could also refine the polyhedral and needle-like phases, but its effect was not as prominent as the addition of cerium
4. Combined addition of 0.03 wt.% Ti and 0.06 wt.% Ce (Ti : Ce = 1 : 3) led to the promotion of comprehensive tensile properties of the Al-Si-Cu-Fe-Mn alloy. In this moment, the UTS, YS values, and the % elongation reached the maximum value. When the ratio of Ti : Ce was increased, the modification, refinement, and strengthening effect were decreased

## Figures and Tables

**Table 1:** Chemical composition of the experimental alloys.

Alloys	Chemical composition (wt.%)									
	Si	Fe	Cu	Zn	Mn	Mg	Ti	C	Ce	Al
Al-Si-Cu-Fe-Mn	10.92	2.12	2.19	0.88	0.51	0.07	0.01	—	—	Bal.
Al-Ti-C	—	—	—	—	—	—	4.95	0.50	—	Bal.
Al-10Ce	—	—	—	—	—		—	—	9.95	Bal.

**Table 2:** Added amounts of titanium and cerium in the experimental alloys.

Sample	#1	#2	#3	#4	#5	#6	#7	#8	#9	#10
Ti (wt.%)	0	0	0	0	0.03	0.06	0.09	0.03	0.06	0.09
Ce (wt.%)	0	0.03	0.06	0.09	0	0	0	0.09	0.06	0.03

**Table 3:** Compositions of secondary phases in the three marked regions in Figure 7(d).

Region	Al	Si	Cu	Fe	Mn	Ce	Ti
(1)	66.94	22.11	1.37	—	—	3.12	6.46
(2)	69.71	13.09	0.02	9.26	7.94	—	—
(3)	58.47	41.53	—	—	—	—	—

## Data Availability

The data used to support the findings of this study have been deposited in the “Characterization of Microstructure and Properties of Recycled Al-Si-Cu-Fe-Mn Alloys with Combined Titanium and Cerium Using Phase Identification Techniques” repository (3472743.v1). The datasets used or analyzed during the current study are available from the corresponding author on reasonable request. The MATLAB programs for microstructures quantification in this research can also be provided in the format of “.m” files. Actually, all results and figures generated or analyzed during this study are included in this published article.

## References

[1] Okayasu M., Ohkura Y., Takeuchi S., Takasu S., Ohfuji H., Shiraishi T. (2012). A study of the mechanical properties of an Al–Si–Cu alloy (ADC12) produced by various casting processes. *Materials Science and Engineering: A*.

[2] Yang L., Li W., du J., Wang K., Tang P. (2016). Effect of Si and Ni contents on the fluidity of Al-Ni-Si alloys evaluated by using thermal analysis. *Thermochimica Acta*.

[3] Yang H., Dong E., Zhang B., Yuan Y., Shu S. (2017). Fabrication and characterization of in situ synthesized SiC/Al composites by combustion synthesis and hot press consolidation method. *Scanning*.

[4] Tang P., Hu Z., Zhao Y., Huang Q. (2017). Investigation on the solidification course of Al–Si alloys by using a numerical Newtonian thermal analysis method. *Materials Research Express*.

[5] Chen H., Yuan D., Wu S., Wang H., Xie W., Yang B. (2017). Relationship between microstructure and properties of Cu-Cr-Ag-(Ce) alloy using microscopic investigation. *Scanning*.

[6] Wan B., Chen W., Liu L., Cao X., Zhou L., Fu Z. (2016). Effect of trace yttrium addition on the microstructure and tensile properties of recycled Al–7Si–0.3Mg–1.0Fe casting alloys. *Materials Science and Engineering: A*.

[7] Han G., Liu X. (2016). Phase control and formation mechanism of Al–Mn(–Fe) intermetallic particles in Mg–Al-based alloys with FeCl_3_ addition or melt superheating. *Acta Materialia*.

[8] Gao T., Li Z. Q., Zhang Y. X., Liu X. F. (2018). Evolution, behavior of *γ*-Al_3.5_FeSi in Mg melt and a separation method of Fe from Al–Si–Fe alloys. *Acta Metallurgica Sinica*.

[9] Elgallad E. M., Ibrahim M. F., Doty H. W., Samuel F. H. (2018). Microstructural characterisation of Al–Si cast alloys containing rare earth additions. *Philosophical Magazine*.

[10] Basak C. B., Babu N. H. (2016). Morphological changes and segregation of *β*-Al_9_Fe_2_Si_2_ phase: a perspective from better recyclability of cast Al-Si alloys. *Materials & Design*.

[11] Guan Y. Q., Du J., Wu T. Q., Cao D. D. I. N. H., Li W.-F., Xu D.-Y. (2017). Effects of B-Cr on microstructure and mechanical properties of 20%Mg2Si/A356-1.3%Fe recycled aluminum matrix composites. *Chinese Journal of Nonferrous Metals*.

[12] Farahany S., Ourdjini A., Idrsi M. H., Shabestari S. G. (2013). Evaluation of the effect of Bi, Sb, Sr and cooling condition on eutectic phases in an Al–Si–Cu alloy (ADC12) by in situ thermal analysis. *Thermochimica Acta*.

[13] Farahany S., Ourdjini A., Abu Bakar T. A., Idris M. H. (2014). A new approach to assess the effects of Sr and Bi interaction in ADC12 Al-Si die casting alloy. *Thermochimica Acta*.

[14] Zhang P., Li Z., Liu B., Ding W. (2016). Effect of chemical compositions on tensile behaviors of high pressure die-casting alloys Al-10Si-*y*Cu-*x*Mn-*z*Fe. *Materials Science and Engineering: A*.

[15] Wang E. R., Hui X. D., Chen G. L. (2011). Eutectic Al–Si–Cu–Fe–Mn alloys with enhanced mechanical properties at room and elevated temperature. *Materials & Design*.

[16] Tang P., Li W., Zhao Y., Wang K., Li W., Zhan F. (2017). Influence of strontium and lanthanum simultaneous addition on microstructure and mechanical properties of the secondary Al-Si-Cu-Fe alloy. *Journal of Rare Earths*.

[17] Song X., Yan H., ZHANG X. (2017). Microstructure and mechanical properties of Al-7Si-0.7Mg alloy formed with an addition of (Pr+Ce). *Journal of Rare Earths*.

[18] Farahany S., Idris M. H., Ourdjini A. (2015). Effect of bismuth and strontium interaction on the microstructure development, mechanical properties and fractography of a secondary Al–Si–Cu–Fe–Zn alloy. *Materials Science and Engineering: A*.

[19] Tang P., Li W., Wang K. (2017). Effect of Al-Ti-C master alloy addition on microstructures and mechanical properties of cast eutectic Al-Si-Fe-Cu alloy. *Materials & Design*.

[20] Jiang F., Zhang H., Meng X., Li L. (2014). Effects of Ti addition on the microstructures and mechanical properties of the Al–Mn–Mg–RE alloy. *Materials & Design*.

[21] Min S., Kanghua C., Lanping H. (2007). Effects of Ce and Ti on the microstructures and mechanical properties of an Al-Cu-Mg-Ag alloy. *Rare Metals*.

[22] Ahmad R., Asmael M. B. A. (2016). Influence of cerium on microstructure and solidification of eutectic Al-Si piston alloy. *Materials and Manufacturing Processes*.

[23] Elgallad E. M., Doty H. W., Alkahtani S. A., Samuel F. H. (2016). Effects of La and Ce addition on the modification of Al-Si based alloys. *Advances in Materials Science and Engineering*.

[24] Nordin N. A., Farahany S., Abu Bakar T. A., Hamzah E., Ourdjini A. (2015). Microstructure development, phase reaction characteristics and mechanical properties of a commercial Al–20%Mg_2_Si–*x*Ce in situ composite solidified at a slow cooling rate. *Journal of Alloys and Compounds*.

[25] Zhang L., Damoah L. N., Grandfield J. F., Eskin D. G. (2013). Current technologies for the removal of iron from aluminum alloys. *Essential Readings in Light Metals: Cast Shop for Aluminum Production, Volume 3*.

[26] Ryan J. A., Botham G. H. (2002). Iron in aluminum alloys. *Analytical Chemistry*.

[27] Li N., Liu X., Wang Q. (2017). Effect of combined addition of Al-Ti-B ribbon and Zr element on the microstructure, mechanical and damping properties of ZA22 alloy. *Materials & Design*.

[28] Qiu C., Miao S., Li X. (2017). Synergistic effect of Sr and La on the microstructure and mechanical properties of A356.2 alloy. *Materials & Design*.

[29] Rao Y., Yan H., Hu Z. (2013). Modification of eutectic silicon and *β*-Al_5_FeSi phases in as-cast ADC12 alloys by using samarium addition. *Journal of Rare Earths*.

[30] Boselli J., Pitcher P. D., Gregson P. J., Sinclair I. I. (1999). Secondary phase distribution analysis via finite body tessellation. *Journal of Microscopy*.

[31] Spowart J. E., Maruyama B., Miracle D. B. (2001). Multi-scale characterization of spatially heterogeneous systems: implications for discontinuously reinforced metal–matrix composite microstructures. *Materials Science and Engineering: A*.

[32] Du J., Ding D., Xu Z. (2017). Effect of CeLa addition on the microstructures and mechanical properties of Al-Cu-Mn-Mg-Fe alloy. *Materials Characterization*.

[33] Wang S.-r., Ma R., Wang Y.-z., Wang Y., Yang L.-y. (2012). Growth mechanism of primary silicon in cast hypoeutectic Al-Si alloys. *Transactions of Nonferrous Metals Society of China*.

[34] Sebaie O. E., Samuel A. M., Samuel F. H., Doty H. W. (2008). The effects of mischmetal, cooling rate and heat treatment on the eutectic Si particle characteristics of A319.1, A356.2 and A413.1 Al–Si casting alloys. *Materials Science and Engineering: A*.

[35] Raghavan V. (2011). Al-Fe-Mn-Si (aluminum-iron-manganese-silicon). *Journal of Phase Equilibria and Diffusion*.

[36] Fan C., Long S., Mingfang W., Huaide Y. (2014). Effect of Ce-rich mischmetal addition on microstructure and tensile properties of secondary Al-Si alloys. *Rare Metal Materials and Engineering*.

[37] Li Q., Xia T., Lan Y., Zhao W., Fan L., Li P. (2013). Effect of rare earth cerium addition on the microstructure and tensile properties of hypereutectic Al–20%Si alloy. *Journal of Alloys and Compounds*.

[38] Yu L., Liu X., Ding H., Bian X. (2007). A new nucleation mechanism of primary Si by like-peritectic coupling of AlP and Al_4_C_3_, in near eutectic Al–Si alloy. *Journal of Alloys and Compounds*.

[39] Lu S. Z., Hellawell A. (1987). The mechanism of silicon modification in aluminum-silicon alloys: impurity induced twinning. *Metallurgical Transactions A*.

[40] Chang J. Y., Kim G. H., Moon I. G., Choi C. S. (1998). Rare earth concentration in the primary Si crystal in rare earth added Al-21wt.%Si alloy. *Scripta Materialia*.

[41] Hegde S., Prabhu K. N. (2008). Modification of eutectic silicon in Al–Si alloys. *Journal of Materials Science*.

[42] Slater J. C. (1964). Atomic radii in crystals. *The Journal of Chemical Physics*.

[43] Ma K., Wen H., Hu T. (2014). Mechanical behavior and strengthening mechanisms in ultrafine grain precipitation-strengthened aluminum alloy. *Acta Materialia*.

[44] Murphy A. M., Howard S. J., Clyne T. W. (1998). Characterisation of severity of particle clustering and its effect on fracture of particulate MMCs. *Materials Science and Technology*.

[45] Gao Q., Wu S., Lü S., Xiong X., Du R., An P. (2017). Effects of ultrasonic vibration treatment on particle distribution of TiB_2_ particles reinforced aluminum composites. *Materials Science and Engineering: A*.

